# Unraveling regulatory divergence, heterotic malleability, and allelic imbalance switching in rice due to drought stress

**DOI:** 10.1038/s41598-021-92938-x

**Published:** 2021-06-29

**Authors:** Nelzo C. Ereful, Antonio Laurena, Li-Yu Liu, Shu-Min Kao, Eric Tsai, Andy Greenland, Wayne Powell, Ian Mackay, Hei Leung

**Affiliations:** 1 John Bingham Laboratory, National Institute of Agricultural Botany (NIAB), 93 Lawrence Weaver Road, Cambridge, CB3 0LE UK; 2 Philippine Genome Center for Agriculture/Plant Physiology Lab, Institute of Plant Breeding, University of the Philippine Los Baños, 4031 Laguna, Philippines; 3 International Rice Research Institute (IRRI), Los Baños, 4031 Laguna, Philippines; 4 Philippine Genome Center for Agriculture, University of the Philippine Los Baños, 4031 Laguna, Philippines; 5 Department of Agronomy, National Taiwan University (NTU), Taipei City 100, Taiwan; 6 Compass Bioinformatics, New Taipei City, Taiwan; 7 SRUC, Peter Wilson Building, West Mains Road, Edinburgh, EH9 3JG UK

**Keywords:** Computational biology and bioinformatics, Genetics, Molecular biology, Plant sciences

## Abstract

The *indica* ecotypes, IR64, an elite drought-susceptible variety adapted to irrigated ecosystem, and Apo (IR55423-01 or NSIC RC9), a moderate drought-tolerant upland genotype together with their hybrid (IR64 × Apo) were exposed to non- and water-stress conditions. By sequencing (RNA-seq) these genotypes, we were able to map genes diverging in cis and/or trans factors. Under non-stress condition, cis dominantly explains (11.2%) regulatory differences, followed by trans (8.9%). Further analysis showed that water-limiting condition largely affects trans and cis + trans factors. On the molecular level, cis and/or trans regulatory divergence explains their genotypic differences and differential drought response. Between the two parental genotypes, Apo appears to exhibit more photosynthetic efficiency even under water-limiting condition and is ascribed to trans. Statistical analyses showed that regulatory divergence is significantly influenced by environmental conditions. Likewise, the mode of parental expression inheritance which drives heterosis (HET) is significantly affected by environmental conditions indicating the malleability of heterosis to external factors. Further analysis revealed that the HET class, dominance, was significantly enriched under water-stress condition. We also identified allelic imbalance switching in which several genes prefer IR64- (or Apo-) specific allele under non-stress condition but switched to Apo- (or IR64-) specific allele when exposed to water-stress condition.

## Introduction

The relative contribution of both regulatory and coding sequences in generating phenotypic variation and thus evolutionary differences and distinct ecological adaptation has been a subject of recent reports. Regulatory sequences, in particular, have been associated with evolutionary and adaptive divergence in humans^1^, *Drosophila*^2–5^, yeast^6^, *Arabidopsis*^7^, tomato^8^, and stickleback^9^. Some of these reports reveal that it is the changes in cis-acting regulatory sequences that contribute predominantly to phenotypic divergence^3,5,7,10^. However, other investigations have claimed that there is an equal contribution of both cis and trans regulatory factors to evolutionary divergence^8,11^.

In investigating regulatory divergence, allele-specific expression (ASE) imbalance or simply allelic imbalance (AI) is assessed in heterozygotes. This happens when one of the two alleles in a hybrid is expressed at levels more significant than the other. Several platforms have been used to analyze ASE including pyrosequencing (e.g. Wittkopp et al*.*^2^) and most recently, RNA-seq. ASE has been a subject of investigations in several organisms including plants (e.g. rice, barley, maize, *Arabidopsis*), model animals (e.g. mouse, *Drosophila*), and humans. In a hybrid, both alleles are exposed to the same nuclear environment, thus asymmetric expression of the hybrid alleles is attributed to cis regulatory polymorphism. On the other hand, total expression difference between two genotypes which cannot be explained by cis-regulatory differences is ascribed to trans^2,4^. Hence, expression differences between the parental genes that disappear in the hybrid are caused by trans effects^6^.

Despite the increasing information of the participation of local and distal regulatory sequences to the expression divergence of organisms, regulatory differences in rice, arguably the world’s most important crop, is poorly understood. Moreover, little is known about the effect of stress as a selective agent on gene regulation. A report on rice demonstrated that regulatory divergence is associated with ecological speciation and adaptive alterations^12^.

A study shows that modern Green Revolution (GR) rice varieties are typically drought-sensitive due to tight linkage between the loci involved in drought tolerance and plant height^13^. Released 50 years ago, IR8, the GR rice is a high-yielding semi-dwarf rice variety, and is derived from a cross between Peta and Dee-geo-woo-gen (DGWG), both of which are *indica*^14^. With a coefficient of parentage (COP) of 0.131 (see pedigrees, Supplementary Fig. S1), IR64 and Apo, the materials used in this study, have descended from IR8. Despite their common ancestry, these genotypes show differential response to drought conditions and contrasting ecological adaptation – IR64 thrives in irrigated ecosystem; Apo, in upland. Understanding their regulatory differences may therefore provide insights on the molecular basis of their varying drought response and contrasting adaptation.

Majority of previous studies explored regulatory divergence between or within two or more interspecifically related organisms (e.g., *Drosophila*, *Arabidopsis*, yeast). In this study, two intra-sub-specifically related rice genotypes (both *indica*) were tested for cis- and trans-regulatory differences on a genome-wide scale. This may shed hints on the resolving power of mRNA-seq to dissect regulatory divergence between highly genetically related inbred lines. Such strategy may enable the identification of loci with divergent regulatory regions and potentially causative polymorphism between closely related genotypes.

Our investigation is limited to one-way specific cross (IR64 × Apo) to provide preliminary information on the regulatory differences between the two rice genotypes grown under normal (well-watered or non-stress) and water-stress conditions; the latter to simulate field drought conditions. Such environmental perturbation may further drive regulatory differences.

## Results and discussion

The genotypes IR64, Apo and their hybrid (IR64 × Apo) were exposed to both non- (control) and water-stress conditions at early flowering stage and were sequenced using paired-end mRNA-seq (3 genotypes × 2 treatments × 2 replicates) (see Materials and Method). We then tested these datasets for evidence of cis and/or trans regulatory differences. Intra-sub-specific comparison of two rice genotypes with contrasting tolerance against water-limiting conditions allows the detection of regulatory differences at candidate loci putatively involved in their distinct ecological adaptation and divergent response against drought. In this paper, “cis and/or trans” means cis, trans, and their interactions which include cis + trans (synergistic), cis × trans (antagonistic), and compensatory.

To be consistent with our previous papers^15,16^ and allow comparison of expression data, we used the *O. sativa* ssp. *japonica* (cv. Nipponbare) MSUv7 cDNA to map the parental reads. Variant calls (SNPs and Indels) between the two inbred parental lines were identified to discern which parent-specific allele belongs to which parental genotype in the F1. We then created a pseudo-reference to map the heterozygote reads and analyzed for cis/trans regulatory divergence. In the hybrid, since both genotype-specific alleles are exposed to the same trans-acting factors, asymmetric expression is attributed to cis differences or allele-specific epigenetic changes^3,17^.

Using this approach, 9141 genes were found to have one or more mapped reads across genotype–line–treatment combinations (Supplementary Table S1). This modest number of genes may be attributed to the genetic relatedness of the two rice ecotypes, both of which are *indica*. Computationally, as variants were used as parent-specific tags, these numbers represent transcript isoforms with SNP-reads heterozygous between the inbred genotypes, thus, heterozygous in the hybrid.

Only genes with a total parental read count of at least 20 in each treatment were considered for further analysis (IR64 + Apo ≥ 20). We computed the expression ratios (log_2_-transformed) of genes between the two parental genotypes (Parental, P = log_2_(IR64/Apo)) and between the two parent-specific alleles in the F1 heterozygotes (Cis, C = log_2_(IR64_F1_/Apo_F1_)) (see Materials and Methods); trans differences are computed as, T = P – C. By plotting the log_2_-transformed expression ratios of the parents (P) against the hybrid (C) we were able to map transcript isoforms diverging in cis and/or trans regulatory factors and estimate their contributions to the total divergence of the two genotypes under non- and water-stress conditions.

If cis-regulatory differences fully explain divergence between the two genotypes, points (i.e. transcript isoforms) will lie closely on the y = x curve (tested by binomial/Fisher’s exact tests, FDR < 0.5%; see “Materials and Methods” for complete statistical tests). On the other hand, if trans-regulatory divergence explains their differences, points (i.e. isoforms) will lie at the y = 0 curve (exact tests, FDR < 0.5%)^2–4^. Additionally, cis + trans (synergistic), cis × trans (antagonistic), and compensatory effects may also explain their differences.

## Regulatory divergence under non-stress conditions

**Figure 1:**
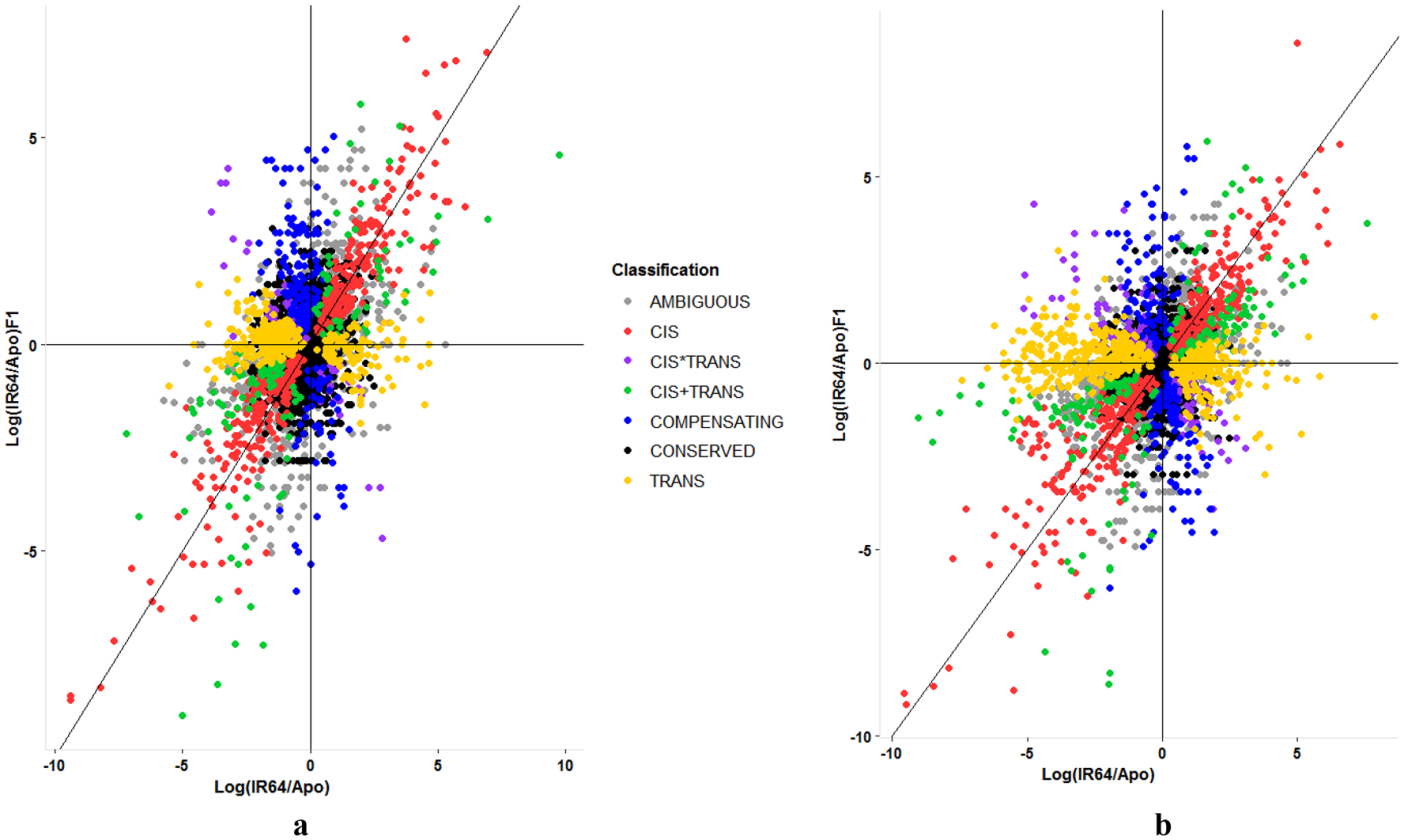
The “scattering” effect of drought stress on cis and/or trans regulatory architecture between two *indica* genotypes: IR64 and Apo under (**a**) non- and (**b**) water-stress conditions. Visual inspection of the two graphs shows more dispersed points of B compared to A. X-axis indicates expression ratios (in log_2_) between the parental genotypes, Log_2_(IR64/Apo); Y-axis, between the two parent-specific alleles in the hybrid, Log_2_(IR64/Apo)F1. Figures generated using ggplot2 (https://ggplot2.tidyverse.org)^23^ in R (v 4.0.2 Comprehensive R Archive Network (CRAN); https://www.R-project.org)^24^.

Under non-stress conditions, 3705 of the 9141 isoforms were found to have a minimum total mapped read of 20 or more between the parents (IR64 + Apo ≥ 20). Of these, 415 (11.2%) and 328 (8.9%) of the 3705 isoforms exhibit cis- and trans-regulatory divergence, respectively, between the parents (Table 1; Fig. 1; see Supplementary Table S2 for a complete list of isoforms diverging in cis and/or trans regulatory factors under non-stress treatment). Apparently, cis dominantly explains the regulatory differences over the other categories between IR64 and Apo (exact tests, FDR < 0.5%). (“Conserved” and “ambiguous” categories were omitted from this comparison). These are shown as red points (i.e. transcript isoforms) lying within or close to the y = x curve (Fig. 1a). GO enrichment analysis of isoforms diverging in cis using AgriGO^18^ showed significant enrichment of genes associated with “response to stress” (biological process, FDR < 0.05; 70 genes; Supplementary Fig. S2) and “nucleolus” (cellular component, FDR < 0.05; 15 genes; Supplementary Fig. S3). The former suggests that their contrasting response against stress is explained by cis divergence. On the other hand, there is now growing evidence of the participation of nucleolus to abiotic stress including drought (reviewed in Kalinina et al*.*^19^).

**Table 1:** Summary of the number of isoforms exhibiting cis and/or trans regulatory divergence between IR64 and Apo under non- and water-stress conditions. χ^2^ statistics (with *P*-values; test for 2 × 2 contingency or two independent proportions with Yates’ continuity correction) indicates the significant difference of the number of isoforms classified in each regulatory category between the two treatments.

Regulatory factors/interactions	Non-stress No. of genes (%)	Water-stress No. of genes (%)	χ^2^ statistics (*P*-value)
cis	415 (11.2)	456 (12.6)	3.19 (0.074)
cis + trans (synergistic)	75 (2.0)	194 (5.4)	56.45 (< 0.001)
cis × trans (antagonistic)	135 (3.6)	118 (3.3)	0.72 (0.397)
compensatory	187 (5.1)	188 (5.2)	0.05 (0.828)
trans	328 (8.9)	551 (15.2)	69.34 (< 0.001)
Ambiguous	696 (18.8)	752 (20.7)	4.315 (0.038)
Conserved	1869 (50.4)	1366 (37.7)	120.529 (< 0.001)
Total	3705	3626

GO analysis of isoforms diverging in trans under non-stress conditions showed significant enrichment of genes associated with “photosynthesis” and “generation of precursor metabolites and energy” (biological process, FDR < 0.05; Supplementary Fig. S4) and “thylakoid” and “plastid” (cellular components, FDR < 0.05; Supplementary Fig. S5). Analysis of the log_2_-transformed expression ratios between the two parental genotypes showed that Apo exhibited higher expression fold change (FC) over IR64 in all of the 21 genes enriched in photosynthesis, 18 of which showed at least 2 × FC. This photosynthetic efficiency of Apo in terms of expression is ascribed to trans and may further account for its tolerance against drought.

Collectively, cis and trans largely explain the divergence of the two genotypes, although compensatory (5.1%), cis + trans (synergistic, 2.0%) and cis × trans (antagonistic, 3.6%) also contribute to these differences, albeit modestly. GO enrichment analysis of genes diverging in cis + trans showed significant association with “response to stress” and “response to stimulus” (biological process; FDR < 0.05) most of which were significantly expressed in the parent Apo (76% or 22 of the 29 genes significantly expressed at FC ≥ 2 ×).

Interestingly, one of the genes diverging in cis + trans is the transcription factor ‘no apical meristem’ (LOC_Os12g29330), identified to be a mainstay in the drought-response region in the Vandana/Way Rarem cross (OsNAM_12.1_)^20^.

In our previous paper^15^, this gene tightly co-localizes with a drought-yield QTL RM511, with an estimated distance of 9 kb. In this paper, it is highly expressed in IR64 as indicated by the positive ratios in both parental (IR64/Apo) and hybrid genotypes (F1_IR64_/F1_Apo_) in both non- and water-stress conditions (Supplementary Table S3). However, while IR64 expresses this TF, it is drought-susceptible. Rice SNP-Seek database^21,22^ confirms the structural differences of the 2 Kb upstream region of this locus between the two genotypes, i.e. alignments show sequence variations (SNPs and InDels) when pairwise-aligned (Supplementary Fig. S6). A multiple intraQTL in the qDTY12.1 region centered on the NAM gene (OsNAM12.1) was reported to provide a concerted drought response^20^. In our study, all genes in the qDTY12.1 region but one (LOC_Os12g29220, nodulin Mt3, OsMtN312.1) co-localizing with the NAM gene were not expressed (Supplementary Table S4). Thus, the expression of this suite of genes in IR64 through genome editing or other conventional platforms may be an interesting area of future study.

*Cis/trans architecture largely explains genotypic differences.* Overall differences between the two parental genotypes were determined using DESeq2^25^, genotypic differential expression or GDE, to capture loci varying between IR64 and Apo across the treatments. Using the 9141 isoforms (described above), GDE between the parents showed 146 genotypically differentially expressed genes (at FC ≥ 2 or |log_2_FC|≥ 1, FDR < 0.05; Supplementary Table S5). Of these GDE genes, 89 (or 61%) exhibited cis divergence between the genotypes; 40, cis + trans (27.3%); 9, trans (6%). Taking all these proportions together, 94% (or 138 of the 146) of the genotypic variations are explained by cis and/or trans regulatory differences between the genotypes. Hence, on the molecular level, cis/trans regulatory landscape explains GDE.

Apparently, local regulatory variations explain most of the genotypic differences. Reports showed that natural selection reduces the level of variability of local genomic regions leaving molecular patterns of selection such as increased genetic divergence around adaptive loci^9,26,27^. Modern selection and recent breeding procedures have likely generated these differences in which most cis/trans regulatory factors and their interactions appear to favor Apo. These features possibly explain their varying ecological adaptations to irrigated (IR64) and upland (Apo) ecosystems and the susceptibility of IR64 and tolerance of Apo to water-limiting conditions.

Our finding is consistent with previous reports which suggest that cis-regulatory changes explain the adaptive and ecological divergence between two species of rice (*O. rufipogon* and *O. nivara*)^12^, between lowland and upland ecotypes of the perennial C_4_ grass, *Panicum hallii*^28^, and among species of *Arabidopsis* (*A. lyrata, A. halleri* and *A. thaliana*)^7^. Furthermore, the finding that cis effects can explain interspecies divergence is common in nature as was previously reported between two subspecies of rice (*indica* and *japonica*)^29^, in *Drosophila* ^2,3,30^, coffee^31^, stickleback^9^, and across *Drosophila mojavensis* populations^32^.

## Regulatory differences under water-limiting conditions

To examine how water-stress treatment changes the regulatory landscape, all lines (parental and hybrid) were exposed to water-limiting conditions. Using the same pipeline as the non-stress treatment, 3626 of the 9141 isoforms were found to have a minimum total mapped read of 20 or more between the parents (IR64 + Apo ≥ 20) under water-stress conditions. Their cis/trans regulatory landscape under water-stress is plotted in Fig. 1B. Initial comparative visualization of the two figures (Fig. 1A vs B) reflects the impact of stress as a mechanism to drive cis/trans regulatory differences. Such change in the molecular landscape is suggestive of the effect of drought as an agent of selection for gene regulation.

Of the 3626 isoforms, 456 (or 12.6% of the 3626) and 551 (15.2%) of genes exhibit cis- and trans-regulatory divergence, respectively, which could explain differences between the two genotypes under stress conditions (Table 1; Fig. 1B; see Supplementary Table S6 for a complete list of genes). These results suggest that under water-stress conditions, trans-acting regulatory variations dominantly explain their differences, superseding cis. The χ^2^ test confirms that it is the trans and cis + trans that are significantly affected by the abiotic stress (Table 1), although trans is more significantly affected (χ^2^ = 69.34) than the latter (χ^2^ = 56.45; *P* < 0.001). Similar to previous findings^9,33,34^, cis is insensitive to environmental perturbations (χ^2^ = 3.19; *P* = 0.074).

The finding that trans regulation differs between environmental regimes agrees with previous studies in yeast^6^ and *Arabidopsis*^33^. We suspect that, as some trans factors are mobile elements (expression; e.g. transcription factors and non-coding RNAs) and can diffuse outside the nucleus, they have been primarily affected by the abiotic stress. Proteins such as TFs have been known to be directly exposed to evolutionary and selection pressures. Additionally, the sensitivity of trans to changes in environmental conditions may be ascribed to its broader mutational target size (sequence) compared to cis mutations^2,35,36^. Similar to non-stress conditions, trans effects under water-limiting conditions showed GO enrichment in “photosynthesis” (Supplementary Fig. S7), “thylakoid” and “plastid” (Supplementary Fig. S8). Apo demonstrates a higher expression fold change over IR64 across all genes enriched in “photosynthesis” (FC ≥ 2, FDR < 0.05; 20 genes). Apo appears to photosynthesize more efficiently even under water-limiting regime and is ascribed to trans. Such pattern is also observed under non-stress condition.

*cis/trans architecture explains differential drought response*. Using 3-way differential expression (referred here as drought DE or DDE), expression variations that arise due to the interactions between the parental genotypes and environment can be detected (G × E) using DESeq2 (see “Materials and Methods”). Results showed that 32 isoforms respond to water stress across genotypes (Supplementary Table S7), 13 of which exhibit trans regulatory differences; 12, cis + trans; 3, cis and; 2, cis × trans. This means that 94% (or 30 of the 32 isoforms) of the drought-responsive genes is explained by cis/trans architecture under water stress. Hence, on the molecular level, the two genotypes’ varying response against drought is explained by cis/trans regulatory differences.

## Heterosis

Recently, evidence of the contributions of cis and/or trans regulation to hybrid vigor has been elucidated^27,36–38^. We, therefore, explored the expression performance of the hybrid relative to its parents in the two contrasting conditions. Pairwise *t*-test was performed between each genotype pair and categorized specific mode of inheritance based on previous reports^27,40,42,43^ after DESeq2 normalization.

Using the 9141 previously shown to have at least 1 mapped read across genotype–line–treatment combinations, a modest number of transcript isoforms can be unambiguously assigned to a specific heterosis category, at *P* < 0.05 (data not shown). This conservative number is presumably attributed to the parents’ genetic relatedness. We, therefore, expanded the *P*-value at 0.10 to allow the inclusion of a wider number of transcript isoforms for heterosis classification: 173 and 238, under non- and water-stress conditions, respectively (see Supplementary Table S8 and S9 for lists of transcript isoforms and their pattern of heterosis classification, under non- and water-stress conditions, respectively). An extreme majority of the transcript isoforms exhibited no differential expression (No DE) in both conditions (Table 2).

**Table 2:** Hybrid performance. Each number corresponds to the number of transcript isoforms in each heterosis category under non- and water-stress conditions.

Class	Description	Non-stress (No.)	Water-stress (No.)
Additive	Hp > F1 > Lp	25	16
Hp het	Hp = F1 > Lp	32***	76 ***
> Hp het	F1 > Hp ≥ Lp	10**	27**
Lp het	Hp > F1 = Lp	98	112
< Lp het	Hp ≥ Lp > F1	8	7
Ambiguous	Varies^†^	463	505
No DE	Hp = F1 = Lp	8505	8398

^†^e.g. Hp > Lp, Hp = F1 = Lp; Hp > F1, Hp = F1 = Lp.

Asterisks indicate binomial exact test (against the null hypothesis of equal proportion) significance level: (**) *P* < 0.01; (***) *P* < 0.0001.

**Figure 2:**
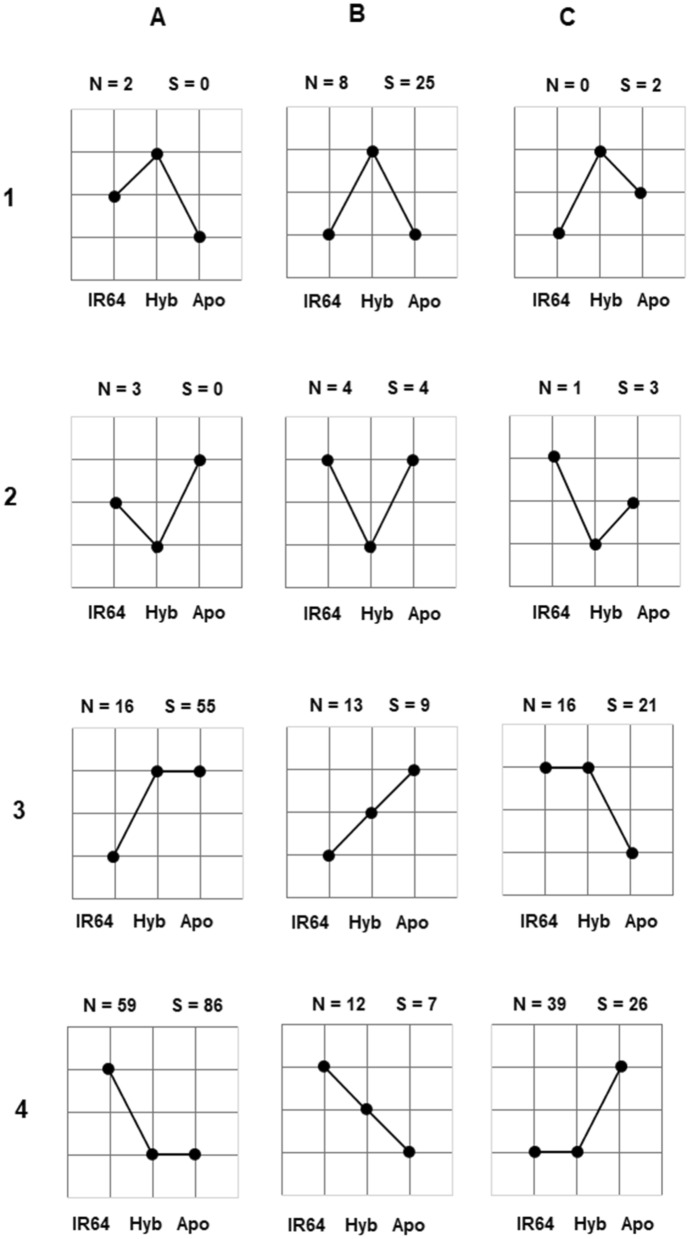
Two-dimensional grid node demonstrating expression performance of the F1 hybrids (Hyb) relative to its parents (Apo and IR64). Values above each panel indicate number of transcript isoforms under unstressed (N) and stressed (S) conditions exhibiting such mode of expression inheritance. Row 1 shows isoforms which exhibits over-dominance; 2, under-dominance; 3A and 4A, paternal (Apo) dominance; 3C and 4C, maternal (IR64) dominance; 3B and 4B, mid-parent or additive.

To easily visualize the mode of expression inheritance, we generated two-dimensional grid nodes indicating the expression performance of the hybrid relative to its parents (Fig. 2; also summarized in Table 2). Results indicated that there is a significant difference in the number of genes exhibiting overdominance between non- and water-stress conditions: 10 and 27, respectively (binomial exact test of equal proportion, *P* < 0.01; Row 1 of Fig. 2; also shown in Table 2). On the other hand, a modest number of transcript isoforms exhibited underdominance under non- and water-stress conditions with 8 and 7, respectively (Row 2; shown in Table 2).

Overall, 141 transcript isoforms exhibited paternal dominance (both high- and low-parent paternal, panels 3A and 4A, respectively); 47, maternal dominance (both high- and low-parent, panels 3C and 4C, respectively) under water-stress conditions. These values are significantly different using binomial exact test of equal proportion (*P* < 0.001). Similarly, paternal dominance (75 isoforms) was favored over maternal (55 isoforms) under unstressed condition, although not significant (binomial, *P* = 0.095). There is an apparent dominance of the paternal (Apo) over maternal allele (IR64) in the hybrid.

We summarized these categories into seven broader classes^28,43^ (Table 2): additive, Hp > F1 > Lp; high-parent heterosis (Hp het), Hp = F1 > Lp; above high-parent het (> Hp het), F1 > Hp ≥ Lp; low-parent het (Lp het), Hp > F1 = Lp; below Lp het (< Lp), Hp ≥ Lp > F1; ambiguous, no clear mode of inheritance pattern; and No DE.

Additive which happens when the expression performance of the hybrid is equal to the combined performance of the parents, showed 25 and 16 isoforms, under non- and water-stress conditions (also shown in panels 3B and 4B of Fig. 2). However, these values are not significantly different (binomial test of equal proportion, *P* = 0.21).

Under non-stress, results indicate the abundance of combined low-parent and below Lp heteroses (Lp het +  < Lp het) as compared to the combined high-parent and above Hp het (Hp het +  > Hp het), with 106 and 42 transcript isoforms (binomial exact test of equal proportion, *P* < 0.0001), respectively. Under water-stress conditions, these values change in which below Lp and low- (< Lp het + Lp het) and high-parent and above Hp (Hp het +  > Hp het) heteroses are approximately balance, 103 and 119, respectively (binomial exact test, *P* = 0.3141). These results suggest the versatile heterotic performance as influenced by the conditions. Above Hp (> Hp het) and high-parent heteroses (Hp het) were observed to increase significantly from non- to water-stress conditions (binomial exact test, *P* < 0.01 and *P* < 0.001, respectively) suggesting elevated expression of several isoforms when exposed to stressful conditions.

*Regulatory divergence, mode of inheritance, environmental conditions and their interactions.* We further explored the relationship between cis and/or trans regulatory divergence to patterns of expression inheritance under both water regimens (Table 3). First, we classified the mode of inheritance into three broad classes^38,39^: additive, dominance, and transgressive. Transgressive mode of inheritance happens when the expression performance of the hybrid is outside the parents’ (illustrated in Rows 1 and 2 of Fig. 2). Then, we assigned and counted the type of cis and/or trans regulatory divergence which the transcript isoform exhibits under each mode of inheritance in both conditions.

**Table 3:** Number of transcript isoforms diverging in cis and/or trans regulatory factors explaining additive, dominance, and transgressive under non- and water-stress conditions.

Regulatory factor^†^	Additive		Dominance		Transgressive	
	Non-stress	Water-stress	Non-stress	Water-stress	Non-stress	Water-stress
cis	11	6	33	43	8	1
trans	1	2	10	43	0	1
cis + trans	5	5	4	31	0	0
cis – trans^‡^	0	0	1	9	0	6

^†^Transcript isoforms exhibiting ambiguous and conserved regulation were excluded from this analysis.

^‡^ To avoid over-dispersion due to ‘0’ values in the succeeding modelling of these counts, opposing or contrasting interactions were combined (antagonistic and compensating).

Initial evaluation of the counts suggests that additive, dominance, and transgressive (at unstressed condition) classes are mostly explained by cis divergence. Using these information (Table 3), results using contingency Pearson's χ^2^ test with simulated *P*-value (based on 1e + 08 replicates; Monte Carlo simulation) showed significant association between regulatory divergence and mode of expression inheritance which drives heterosis (χ^2^ = 36.56, *P* = 2.22e-05). This aligns with a recent paper in which regulatory mechanism during cotton domestication is associated with mode of inheritance^38^. Regulatory divergence was also found to be significantly associated with the changes in water regimen (χ^2^ = 28.29, *P* = 2.42e-06). This suggests that changes in environmental conditions play a significant role in regulatory changes. Recent studies have shown that regulatory divergence was effected by abiotic stresses^28,33^. Interestingly, environmental conditions were also found to be significantly associated with the mode of expression inheritance (χ^2^ = 11.96, *P* = 0.0021), which we further explored below.

The count data (Table 3) was modelled with Poisson (with a log-link function) on regulatory divergence (REG), heterosis (HET), environmental conditions (ENV), and their interactions using a generalized linear model (GLM) (see Materials and Method). Analysis of Deviance using ANOVA function showed that the most suitable model was the full 2-way interactions with single terms (i.e., REG + HET + ENV + REG:HET + REG:ENV + HET:ENV) (*R*^2^ = 0.98; Residual Deviance/df ~ 1), with all interaction terms in the model being significant at either *P* < 0.001 or *P* < 0.01 (see Table 4 for Likelihood Ratio Test (LRT) analysis in R and Supplementary Information 1, for complete Analysis of Deviance table).

**Table 4:** Analysis using LRT showed significant association between two terms of the three factors tested.

Single term deletions					
Model: COUNT ~ REG + HET + ENV + REG:HET + REG:ENV + HET:ENV					
	Df	Deviance	AIC	LRT	Pr(> Chi)
<none>		6.355	108.73		
REG:HET	6	39.014	129.39	32.659	1.2E-05***
REG:ENV	3	35.419	131.8	29.063	2.2E-06***
HET:ENV	2	17.011	115.39	10.655	0.00486**

Signif. codes: ‘***’ 0.001 ‘**’ 0.01.

There appears to be significant interaction between factors tested which is consistent with the results obtained from the Pearson’s χ^2^ test above. This further confirms that the mode of expression inheritance which propels heterotic variability was found significantly associated with changes in environmental conditions (HET:ENV, *P* < 0.01). This further underscores the malleability of heterosis as it is influenced by external factors, suggesting it is not a fixed genetic attribute. Early and recent studies revealed that environment can influence heterosis^40,41^. Further results showed that dominance (a HET class) is significantly enriched under environmental stress (*P* < 0.01; Supplementary Information 1), which is apparent in Table 3.

In summary, these findings suggest that regulatory divergence and environmental conditions are highly associated with heterosis.

## Test of ASE reveals allelic imbalance switching

**Figure 3:**
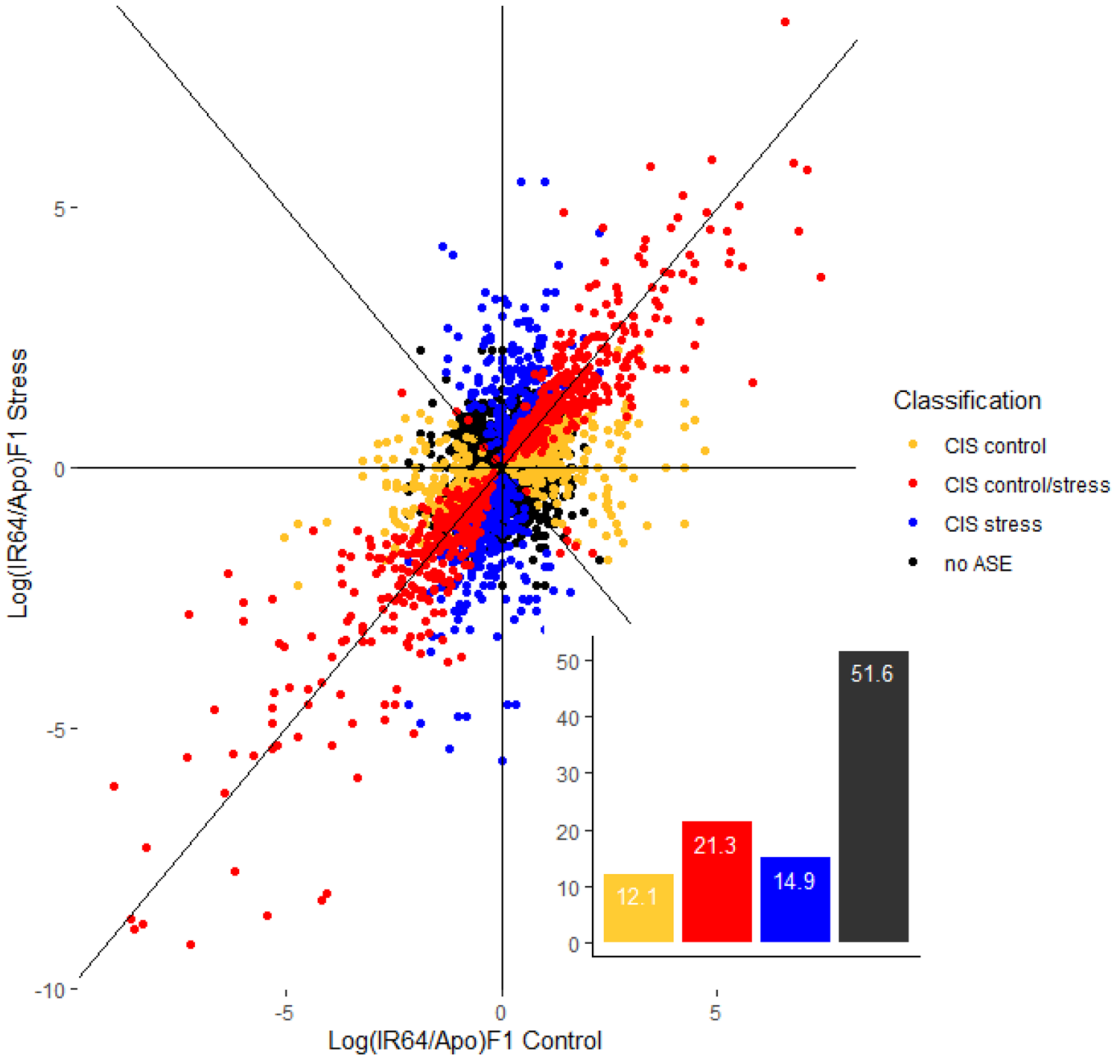
Relative expression ratios between IR64- and Apo-specific alleles in the F1 (Log_2_IR64_F1_/Apo_F1_) under non- (x-axis) and water-stress (y-axis) conditions. Genes are classified based on their CIS category under non- and water-stress conditions (at FDR < 0.5%). Inset bar chart: relative proportion (in percentage) of each CIS category including genes with no ASE classification. Figure generated using ggplot2 (https://ggplot2.tidyverse.org)^23^ in R (v 4.0.2 CRAN; https://www.R-project.org)^24^.

In the previous tests above, we showed the cis and/or trans regulatory landscape of the two parental genotypes and their hybrid under two contrasting conditions. As allelic imbalance is ascribed to cis differences in a heterozygote state, we further assessed the effect of water-limiting conditions on asymmetric expression independent of the parents. This entails plotting the calculated log_2_-transformed expression ratios of the genotype-specific alleles of the hybrid under non-stress or control (x-axis) and water-stress (y-axis) (with binomial exact test at FDR < 0.5%, against the null hypothesis of having no ASE). This gives information on which isoforms exhibit allelic imbalance under non- and/or water-stress conditions in the hybrid. Results are depicted in Fig. 3 (see Supplementary Table S10 for complete list of genes).

Of the 3058 genes that satisfied our criteria (see Materials and Method), 651 isoforms or 21.3% exhibit AI under both conditions (binomial exact test, FDR < 0.5%; red dots in Fig. 3); 371 isoforms or 12.1% (yellow dots), under non-stress; 457 or 14.9% (blue), under stress conditions. In summary, almost half of the genes (48.4%) in the hybrids exhibited ASE imbalance across the two conditions (at FDR < 0.5%). However, these proportions are influenced by FDR levels. 1988 of the 3058 (or 65%) are asymmetrically expressed at FDR < 5% (Supplementary Fig. S9; Supplementary Table S11). Hence, ASE imbalance as a consequence of cis divergence between the two *indica* genotypes is pervasive between the two closely related genotypes. In this study, asymmetric expression was found to be more prevalent under water-limiting compared to non-stress conditions (in both FDR levels).

IR64-specific allele is favorably expressed in the hybrid over Apo-specific allele under non-stress conditions with 133 and 95 isoforms (binomial exact test for equal proportion, *P* < 0.05), respectively, exhibiting ASE imbalance or cis divergence (includes CIS control only; excludes CIS control/stress). However, under water-limiting conditions, Apo-specific allele is favorably expressed over IR64-specific allele with 157 and 134 isoforms, respectively, exhibiting cis divergence (includes CIS stress only; excludes CIS control/stress). These values, however, are not statistically different using binomial exact test for equal proportion (*P* = 0.20).

We expect that all CIS control/stress genes to lie on the y = x curve. However, surprisingly, 11 of these genes lie on the y = –x axis in Fig. 3 (shown as red dots on this curve; FDR < 0.5%). These are transcript isoforms that switch from IR64- to Apo-specific allele (or vice-versa) between well-watered and water-stressed conditions. We call such genes to exhibit allelic imbalance switching in the hybrid. At an FDR < 0.5%, five of the 11 transcript isoforms switch from Apo- to IR64-specific allele; six, from IR64- to Apo-specific allele from non- to water-stress, respectively. On the other hand, there are 31 transcript isoforms exhibiting allelic imbalance switching (at an FDR < 5% with at least 2 × FC in either or both of the conditions). Of these, 10 transcript isoforms switch from Apo- to IR64-specific allele; 21, from IR64- to Apo-specific allele from non- to water-stress, respectively. Apparently, two-thirds of the genes transitioned to the more drought-tolerant allele at water-limiting condition, a rather ingenious mechanism to adjust with the stress (see Supplementary Table S12 for the list of transcript isoforms and their putative functions at both FDR levels).

This bidirectional ‘swinging’ expression behavior between the two co-residing genomes suggests that for some genes, preferential expression is condition-dependent in highly heterozygous organisms. Using the 3 K rice resequencing project^45^, pairwise alignment of the promoter region (2 Kb upstream of the transcriptional start site) of these 11 genes (FDR < 0.5%) between the two parental genotypes confirmed their structural differences (see Supplementary Information 2, stored in github container due to a number of files generated; further described below). However, epigenetic variations may also participate in such attribute. This, however, is an interesting area of further inquiry.

In summary, as hybrids host two enormous genomes, they are endowed with transcriptional versatility. Changes in cis and/or trans regulatory divergence, mode of parental expression inheritance which drives heterotic variability, and allelic imbalance switching all point out to the dynamic transcriptional re-programming of a hybrid when exposed to stressful conditions.

## Materials and methods

### Dry-down experiment

**Figure 4:**
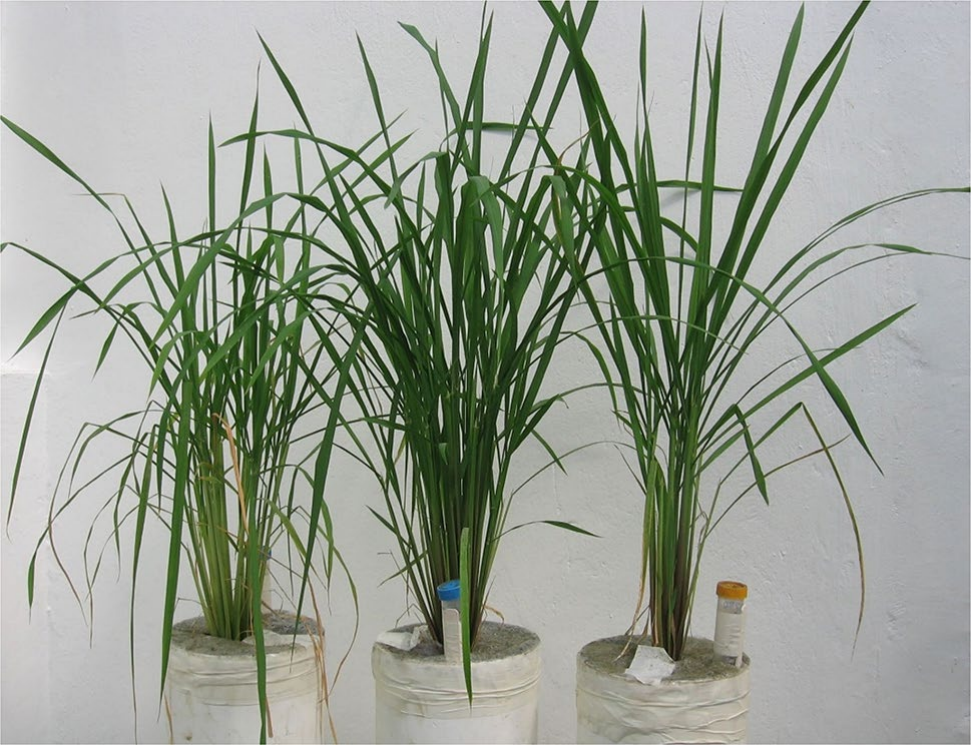
Samples of the rice genotypes IR64 (left), Apo (right), and their F1 hybrid (center) were grown in large pots and were exposed to both non- and water-stress conditions.

Seeds from both parental and hybrid genotypes were initially grown in petri plates under room temperature. After germination, seedlings were transferred to small pots under controlled conditions inside the phytotron, at the International Rice Research Institute (IRRI). Leaf samples were collected to test hybrid status using SSR markers (RM269, RM 511 and RM 80). Hybrid which exhibited clear polymorphism between the parents using these markers were selected for further experiment. Twenty-four (24) seedlings with confirmed polymorphism were transplanted in large pots (see Fig. 4 for sample plants).

Plant samples were exposed to either non- and water-stress conditions. For water-stressed samples, the fraction of transpirable soil water (FTSW) dry-down approach was implemented as previously described^46–48^. Water-limiting conditions was applied by initiating a soil dry-down protocol starting 10 days before heading until the plants reached 0.5 FTSW. To ensure homogeneity of stress applied and that the stress level was reached, all pots were weighed daily.

### RNA extraction

At the end of the dry-down treatment, leaf samples from each plant were collected. Samples were snap-frozen in liquid nitrogen and stored at -80 °C. RNA was extracted using the TRIzol method according to the instructions provided by the supplier (Invitrogen, San Diego, Calif., USA). RNA-seq libraries were prepared as described in Illumina’s standard protocol for RNA-seq using the parental (IR64 and Apo) and F1 RNA samples from each treatment (non- and water-stress).

### Bioinformatics pipeline

All custom PERL, R scripts, Linux commands and Supplementary Information 2 are stored in https://github.com/nelcaster7/regulatory_divergence.

Quality checking. Libraries were sequenced on Illumina GAIIx, generating 38- and 90-base paired end (PE) reads, for the first and second sequencing protocols, respectively. Each sequencing protocol also corresponds to first and second biological replicates. To ensure these dissimilar sizes would not affect the downstream analysis, correlation matrix was generated. Correlations showed mostly 0.9 and 1 between reps 1 and 2 of the same genotype–treatment (two of the 16 sample–replicates showed a correlation of 0.7 presumably due to a number of loci with 0 read counts and/or segments with extremely high read counts which were corrected at pre-filtering and count normalization stages) (Supplementary Fig. S10). We also tested our datasets using Poisson test (“poisson.test” in R) as noises in a sequencing experiment should be Poisson distributed (see github container on Poisson test).

Quality checking was performed using FASTQC; trimming using FASTX-Toolkit (http://hannonlab.cshl.edu/). We checked the 75% percentile of the sequencing qualities for each base of the PE reads in each sample replicate. Bases with 75% percentile of the sequencing qualities < 28 were removed for all reads in the replicate.

#### Creating a consensus pseudo-reference

Our bioinformatics approach employed the construction of an in silico transcriptome pseudo-reference sequence. This strategy is similar to a previous study^49^ which overcomes read-mapping biases which may distort ASE estimation^50^.

The *O. sativa* ssp. *japonica* (cv. Nipponbare) MSU v7 cDNA (http://rice.plantbiology.msu.edu/) was indexed using bowtie2^51^. Using the same tool, reads from IR64 and Apo were separately mapped against the indexed reference sequence (no-mixed; no discordant). Alignment options were set at lenient parameters to allow higher percentage alignment since the materials (IR64 and Apo) are *indica* and the reference is a *japonica*.

Using SAMtools^52^, variants (SNPs and Indels) were identified between the MSU7 reference cDNA sequence and the sorted alignment files of both genotypes (commands: sort and mpileup). Custom PERL scripts were created to call SNPs and InDels common to Apo and IR64 sequences (*indica*), but different from the Nipponbare reference sequence. SNP reads must have at least 5 × coverage and the SNP proportion must be more than 0.8. For InDels, read coverage must also be at least 5 × but InDel proportion should be at least 0.5. We then developed another PERL script which incorporates these variants (SNP and Indels common to IR64 and Apo but different from the MSUv7 cDNA) to modify the reference sequence.

We further mapped 93–11 *indica* RNAseq reads from BGI (http://rise2.genomics.org.cn/) against the pseudo-reference and MSU reference transcriptome sequence and found an increase percentage alignment of 1.09%.

#### SNP calling

Bowtie2 was used to map Apo, IR64 and the F1 genotypes to the pseudo-reference. SAMtools (options: sort and mpileup) was used to sort and parse the mapping results. To find the SNPs between Apo and IR64 sequences, we created a separate PERL script. The criteria, however, should be: SNP reads must have at least 3 × read coverage and SNP proportion must be more than 0.8. We used these SNPs to discern which read belongs to which genotype in the F1. These variant calls allow us to distinguish and quantify the two genotype-specific SNP-reads in the heterozygote. Reads with multiple SNPs may cause over-estimation of reads. To avoid such scenario, we used a read-wise approach by counting the number of reads with SNPs regardless of their frequency within a read. A separate custom PERL script was created to carry out this step.

#### Gene expression estimation and normalization procedure

We counted the number of reads from the parents and the genotype-specific alleles in the F1 using eXpress^53^. All statistical analyses in this study were performed using the software R (v 4.0.2 CRAN)^24^.

Normalization was performed using DESeq2^25^. Normalized count data were rounded off to the nearest integer for downstream statistical tests such as binomial exact test. Average of the normalized read counts of the two replicates for each sample was calculated. Only genes with a total average normalized read count of 20 of the parental samples (IR64 + Apo ≥ 20) were considered for further analysis as recommended^4,54,55^. In cases where an isoform has zero read count in either of the parents (IR64 = 0 or Apo = 0) or in either of the parent-specific allele in the hybrid (F1_IR64_ = 0 or F1_Apo_ = 0), we adjusted 0 to 1 in order to calculate ratios needing positive integers (e.g. log_2_ transformation). The parental read sum however should be at least 20.

#### Cis/trans regulatory differences assignment

Normalized read counts of the parental and the hybrid datasets were analyzed for evidence of regulatory divergence using binomial exact tests followed by FDR analysis^56^ at two significant thresholds of 0.5% and 5% FDR. We did not observe any changes in the general trends between the two FDR levels, e.g. dominance of cis followed by trans under non-stress; and dominance of trans followed by cis under water-stress conditions, both of which were consistent at both FDR levels. The most conservative analysis (i.e. FDR < 0.5%) was finally considered in the Results and Discussions consistent with other papers^4,54^.

Genes significantly expressed in either parental or hybrid genotypes were further analyzed for trans effects by comparing the genotype-specific mRNA abundance in the parents and in the hybrid samples using Fisher’s exact tests followed by FDR analysis at a significant threshold of 0.5%. Regulatory divergence types are classified according to the categories described below. Such analysis and regulatory divergence assignments are adapted from previous studies^2,3,31,54,57^. (R scripts for binomial and Fisher exact tests and regulatory assignments are stored in our github container: https://bit.ly/3tAGGMc).

We then performed χ^2^ test (at *P* < 0.05) on our data count between the two contrasting treatments to determine regulatory categories responding significantly with respect to environmental changes. The analysis was performed using 2-sample test for equality of proportions with Yates’ continuity correction. The number of isoforms in each regulatory category from each condition constitute the first vector; the total number of isoforms in each condition detected, the second.

DESeq2 multi-factor design was used to test for GDE and DDE. We used the raw data count of 9141 genes expressed with at least one mapped read across genotype – line – treatment to test for DE (Table S2). Only the parental read counts were included for this analysis.

GDE was estimated between the genotypes across the two conditions: (IR64 non-stress, IR64 stressed) vs (Apo non-stress, Apo stressed). Raw read counts were used as the input data count with “IR64” and “Apo” as genotypes. Size factors and dispersions were estimated by DESeq2. On the other hand, differences that arise between the two genotypes under drought conditions relative to non-stress (3-way DE or DDE) were also estimated using the model: (IR64_con, Apo_con) vs (IR64_stress) vs (Apo_stress). Using the function “relevel” of DESeq2, we declared “Apo” and “control (non-stress)” as first level parent and condition, respectively. Results were compared to the genes exhibiting cis/trans regulatory divergence under non- and water-stress conditions, respectively, to find any overlaps.

We used AgriGO^58^ to perform Gene Ontology (GO) enrichment analysis located at http://systemsbiology.cau.edu.cn/agriGOv2/, implementing Singular Enrichment Analysis (SEA) with *O. sativa* MSU v7 as the reference background.

#### Binomial exact test between the two genotype-specific alleles in the hybrids

In our previous paper^15^ we have analyzed allelic imbalance in the hybrids in the two treatments separately. Due to recent developments in computational analysis, we have included in this paper a re-analysis of the relative IR64/Apo log-transformed accumulation ratios in the F1 hybrids combining the two treatments in one graph (depicted in Fig. 3). This approach leads to a more robust quantification of ASE imbalance and more intuitive identification of allelic imbalance switching.

A binomial exact test between the two parent-specific alleles in the heterozygote under non- and water-stress conditions was performed using R. This was followed by FDR analysis at a significant threshold of 0.5% (results using FDR < 5% were also shown), which aligns with a previous study^33^. An isoform should have a total read count (average normalized read counts of parent-specific alleles) of 20 or more (F1_Apo_ + F1_IR64_ ≥ 20) in one or both conditions. Isoforms with a total read count of 0 in one or both conditions are discarded. Their log-transformed ratios could not be calculated and binomial exact test could not be performed.

#### Statistical analysis on regulatory divergence, heterosis, environmental conditions and their interactions

We first looked into interactions using contingency Pearson's χ^2^ test between terms or factors with simulated *P*-value (based on 1e + 08 replicates; Monte Carlo simulation) using the data count (Table 3).

We also fit a GLM, Poisson regression with log-link function in R. Here we model the counts (Table 3) as response variable on regulatory divergence (REG: cis, trans, cis + trans, cis – trans), mode of inheritance which drives heterosis (HET: additive, dominance, and transgressive) and environment (ENV: non- and water-stress conditions).

Deviance was done using ANOVA which implements the Analysis of Deviance. The full model (effectively the base error; REG*HET*ENV) interaction was overfit. We successively look at a reasonable fit until all terms in the model are very significant and the residual deviance is not significant. ‘manyglm’ function with Poisson regression in R was used to confirm the AIC value of 108.7. ‘drop1’ function in R with likelihood ratio test (LRT) showed all interaction terms to be significant.

#### Pairwise-comparison of the promoter regions between Apo and IR64

LOC_Os12g29330 (NAM), an established drought-response gene^20^, and 11 transcript isoforms exhibiting allelic imbalance switching were studied for differences in promoter sequence (2 K upstream region). We used the published genomes of both Apo and IR64 available from 3 K re-sequencing project^45^. To confirm cis regulatory divergence between the two genotypes, in silico promoter sequence analysis was performed (Supplementary Information 2 stored in github container: https://bit.ly/32eFZw4).

### Ethical standard on the use of plant materials

We comply to the highest international, national and institutional ethical standards on the use of plant materials. Rice genotypes used in this study are not endangered nor at risk of extinction. Therefore, there are no ethical implications associated with the use of such biological materials.

## Supplementary Information

Supplementary Information 1.

Supplementary Information 2.

Supplementary Information 3.

Supplementary Information 4.

Supplementary Information 5.

Supplementary Information 6.

Supplementary Information 7.

Supplementary Information 8.

Supplementary Information 9.

Supplementary Information 10.

Supplementary Information 11.

Supplementary Information 12.

Supplementary Information 13.

Supplementary Information 14.

Supplementary Information 15.

Supplementary Information 16.

Supplementary Information 17.

Supplementary Information 18.

Supplementary Information 19.

Supplementary Information 20.

Supplementary Information 21.

Supplementary Information 22.

Supplementary Information 23.

Supplementary Information 24.

## Data Availability

All sequencing data from this work are available at NCBI Sequence Read Archive with a submission entry: SUB1568816 with BioProject ID PRJNA338445 and are all located at https://www.ncbi.nlm.nih.gov/sra?term=SRP081221.
