# Author Correction: Unraveling regulatory divergence, heterotic malleability, and allelic imbalance switching in rice due to drought stress

**DOI:** 10.1038/s41598-021-96673-1

**Published:** 2021-08-24

**Authors:** Nelzo C. Ereful, Antonio Laurena, Li‑Yu Liu, Shu‑Min Kao, Eric Tsai, Andy Greenland, Wayne Powell, Ian Mackay, Hei Leung

**Affiliations:** 1 John Bingham Laboratory, National Institute of Agricultural Botany (NIAB), 93 Lawrence Weaver Road, Cambridge, CB3 0LE UK; 2 Philippine Genome Center for Agriculture/Plant Physiology Lab, Institute of Plant Breeding, University of the Philippine Los Baños, 4031 Laguna, Philippines; 3 International Rice Research Institute (IRRI), Los Baños, 4031 Laguna, Philippines; 4 Philippine Genome Center for Agriculture, University of the Philippine Los Baños, 4031 Laguna, Philippines; 5 Department of Agronomy, National Taiwan University (NTU), Taipei City, 100 Taiwan; 6 Compass Bioinformatics, New Taipei City, Taiwan; 7 SRUC, Peter Wilson Building, West Mains Road, Edinburgh, EH9 3JG UK

Correction to: *Scientific Reports* 10.1038/s41598-021-92938-x, published online 29 June 2021

The original version of this Article contained an error in the Heterosis section, where the reference citations were incorrect.

“Recently, evidence of the contributions of cis and/or trans regulation to hybrid vigor has been elucidated^27,36–38^. We, therefore, explored the expression performance of the hybrid relative to its parents in the two contrasting conditions. Pairwise *t*-test was performed between each genotype pair and categorized specific mode of inheritance based on previous reports^27,40,42,43^ after DESeq2 normalization.”

now reads:

“Recently, evidence of the contributions of cis and/or trans regulation to hybrid vigor has been elucidated^28,37–39^. We, therefore, explored the expression performance of the hybrid relative to its parents in the two contrasting conditions. Pairwise *t*-test was performed between each genotype pair and categorized specific mode of inheritance based on previous reports^28,42–44^ after DESeq2 normalization.”

The original Article has been corrected.

