# Supplementary material for: Unraveling regulatory divergence, heterotic malleability, and allelic imbalance switching in rice due to drought stress
Source: Sci Rep. 2021 Jun 29;11:13489. doi: 10.1038/s41598-021-92938-x (PMC8241847; doi:10.1038/s41598-021-92938-x)
Supplement: Supplementary file 1 — Supplementary Information 1. [file 41598_2021_92938_MOESM1_ESM.docx]

**Supplementary Materials**

Suppl. Information 1. Calculating GLM (Poisson) in R to identify significant interaction among REG, HET and ENV

Suppl. Information 2. Pairwise alignment between 2Kb upstream region (promoter region) of 11 transcript isoforms exhibiting allelic imbalance switching (stored in <https://bit.ly/32eFZw4>)

Suppl. Fig. S1. Pedigrees of IR64 and Apo, taken from IRIS.

Suppl. Fig. S2. Graphical results of GO enrichment analysis (biological process) using cis-diverging genes under non-stress conditions as input sequence.

Suppl. Fig. S3. Graphical results of GO enrichment analysis (cellular component) using cis-diverging genes under non-stress conditions as input sequence.

Suppl. Fig. S4. Graphical results of GO enrichment analysis (biological process) using trans-diverging genes under non-stress conditions as input sequence.

Suppl. Fig. S5. Graphical results of GO enrichment analysis (cellular component) using trans-diverging genes under non-stress conditions as input sequence.

Suppl. Fig. S6. Pairwise-alignment results using BLAST between IR64 (CSHL; SNP-seek) and Apo (SNP-seek) of the promoter region for the gene, No apical meristem (LOC_Os12g29330)

Suppl. Fig. S7. Graphical results of GO enrichment analysis (biological process) using trans-diverging genes under water-stress conditions as input sequence.

Suppl. Fig. S8. Graphical results of GO enrichment analysis (cellular component) using trans-diverging genes under water-stress conditions as input sequence.

Suppl. Fig. S9. Relative expression ratios between IR64- and Apo-specific alleles in the F1 (Log_2_IR64_F1_/Apo_F1_) under non- (x-axis) and water-stress (y-axis) conditions (at FDR<5%).

Suppl. Fig. S10. Correlation matrix among genotype–treatment–replicate using normalized read counts (Legend: H, hybrid; numbers succeeding each term represent replicates 1 and 2; c, control, s, stress)

Suppl. Table S1. List of isoforms and their corresponding raw read counts and average normalized read counts which have a minimum mapped read of one or more for at least one column across genotype–line–treatment combinations.

Suppl. Table S2. Isoforms identified to have at least 20 mapped reads in either of the parental genotypes under non-stress conditions and their regulatory divergence classification under non-stress condition

Suppl. Table S3. Expression ratios of the parents and hybrids of LOC_Os12g29930 NAM gene under non- and water-stress

Suppl. Table S4. Gene colocalizing with LOC_Os12g29930 NAM under non- and water-stress conditions. (Note: a table summarizing the genes which co-localize with LOC_Os12g29930, the NAM gene, is included in this Suppl. Table and was taken from the study by Dixit et al. 2015).

Suppl. Table S5. Results of GDE showing isoforms genotypically differentially expressed (FC≥2; FDR<0.05)

Suppl. Table S6. Isoforms identified to have at least 20 mapped reads in either of the parental genotypes under water-stress conditions and their regulatory divergence classification under water-stress condition.

Suppl. Table S7. Results of 3-way differential expression or drought DE (DDE) using DESeq2.

Suppl. Table S8. Heterosis classification using *t*-test at non-stress condition

Suppl. Table S9. Heterosis classification using *t*-test at water-stress condition

Suppl. Table S10. List of transcript isoforms exhibiting ASE, hybrid test implemented using binomial exact test (FDR<0.5%). Isoforms were classified as cis-control, cis-stress, or cis (control/stress).

Suppl. Table S11. List of transcript isoforms exhibiting ASE, hybrid test implemented using binomial exact test (FDR< 5%). Legend, similar to Suppl. Table S10.

Suppl. Table S12. Transcript isoforms exhibiting allelic imbalance switching at 0.5% and 5% FDR
