# Supplementary material for: Unraveling regulatory divergence, heterotic malleability, and allelic imbalance switching in rice due to drought stress
Source: Sci Rep. 2021 Jun 29;11:13489. doi: 10.1038/s41598-021-92938-x (PMC8241847; doi:10.1038/s41598-021-92938-x)
Supplement: Supplementary file 2 — Supplementary Information 2. [file 41598_2021_92938_MOESM2_ESM.docx]

**Pedigree tree for IR 64**

PNO NAME AND LEVEL DSTEP TERM GID

1 IR 64 3 50533

5 +-IR 5657-33-2-1 3 24486

10 | +-IR 5236 0 8180

18 | | +-IR 2006-P3-31-3 3 8082

33 | | | +-IR 1870 0 3299

62 | | | | +-IR 1103-15-8-4-2-2-3 6 < 3290

63 | | | | +-IR 1163-124-1-3 3 < 3298

34 | | | +-IR 1614 0 2723

64 | | | +-IR 22 2 < 2722

65 | | | +-IR 1402 0 < 2201

19 | | +-IR 2146-68-1 2 8111

37 | | +-IR 773 A 1-36-2-1 4 3411

70 | | | +-IR 8 2 < 715

71 | | | +-IR 238-28-3-2 3 < 1127

38 | | +-IR 1916 0 3414

72 | | +-IR 773 A 1-36-2-1 4 * 3411

73 | | +-IR 1915 B 0 < 3413

11 | +-IR 5338 0 8307

20 | +-IR 2061-465-1-4 3 * 8301

16 | | +-IR 833-6-2-1-1 4 3707

26 | | | +-IR 262-43-8-11 3 < 1171

27 | | | +-GAM PAI 30-12-15 3 109

55 | | | >GAM PAI ? # 253220

17 | | +-IR 2040 0 3661

28 | | +-IR 1561-149-1 2 < 3660

29 | | +-IR 1737 0 < 3006

21 | +-IR 2055-475-2 2 7326

46 | +-BPI 121-407 ? 3680

78 | | +-FORTUNA 1 421581

135 | | +-PA CHIAM ? # 420345

79 | | +-SERAUP BESAR 15 ? # 320386

47 | +-IR 1833 0 3199

80 | +-IR 1416-131-5 2 < 3161

81 | +-IR 22 2 < 2722

6 +-IR 2061-465-1-5-5 4 11072

16 +-IR 833-6-2-1-1 4 3707

26 | +-IR 262-43-8-11 3 1171

51 | | +-PETA ? 11

83 | | | +-CINA ? # 168602

84 | | | +-LATISAIL 1 168603

140 | | | >LATISAIL ? # 2271264

52 | | +-IR 95 0 155

85 | | +-PETA ? * 11

86 | | +-IR 86 0 < 138

27 | +-GAM PAI 30-12-15 3 109

55 | >GAM PAI ? # 253220

17 +-IR 2040 0 3661

28 +-IR 1561-149-1 2 3660

58 | +-IR 579-48-1-2 (NILO 11) 3 2583

90 | | +-IR 8 2 < 715

91 | | +-TADUKAN 2 815

144 | | >TADUKAN ? # 2271990

59 | +-IR 747-B2-6-3 4 2577

96 | +-TKM 6 ? < 441

97 | +-IR 746 A 0 < 1076

29 +-IR 1737 0 3006

60 +-IR 24 3 2444

101 | +-IR 8 2 < 715

102 | +-IR 127-2-2 2 < 960

61 +-IR 1721 0 2983

103 +-IR 24 3 * 2444

104 +-IR 1704 0 < 2933

NAME - ">" indicates germplasm derived from germplasm in the line above

"+" indicates generative progenitor of the germplasm above the "+"

female progenitors are above male progenitors

DSTEP - Number of derivative steps from generative source

TERM - Termination code:

"*" indicates lines expanded elsewhere in the tree

"<" indicates lines which could be further expanded

"#" indicates germplasm with unknown parents

**PEIGREE TREE for APO**

PNO NAME AND LEVEL DSTEP TERM GID

1 IR 55423-01 (NSIC RC 9) 2 204538

4 +-UPL RI 5 ? 406626

8 | +-SIGADIS (AICRIP) ? 386533

17 | | +-BLUEBONNET ? 96089

33 | | | +-REXORO 2 38864

50 | | | +-MARONG PAROC ? # 420278

34 | | | +-FORTUNA 2 482

52 | | | >PA CHIAM ? # 420345

18 | | +-BENONG ? 16717

35 | | >BENONG ? # 2891665

9 | +-BPI 76-1 ? 227416

23 | +-FORTUNA 1 421581

36 | +-PA CHIAM ? # 420345

24 | +-SERAUP BESAR 15 ? # 320386

5 +-IR 12979-24-1 (BROWN) 3 67816

13 +-IR 10125 0 14281

25 | +-IR 8838 0 12756

37 | | +-IR 3179-25 2 12242

55 | | | +-IR 1721-11-8-3-2-3 5 < 5495

56 | | | +-C 4-63 ? < 3695

38 | | +-K PATONG 2 9175

58 | | >K PATONG ? # 33206

26 | +-IR 26 2 7845

41 | +-IR 24 3 2444

62 | | +-IR 8 2 * 715

63 | | +-IR 127-2-2 2 < 960

42 | +-TKM 6 (ACC 237) ? 420745

67 | +-CO 18 2 421846

93 | +-VELLAIKAR ? # 421187

68 | +-GEB 24 1 < 385404

14 +-IR 879-314-2 2 8771

29 +-IR 8 2 715

45 | +-PETA ? 11

70 | | +-CINA ? # 168602

71 | | +-LATISAIL 1 168603

96 | | >LATISAIL ? # 2271264

46 | +-DEE GEO WOO GEN 2 2

73 | >DEE GEO WOO GEN ? # 2285932

30 +-IR 759 0 1089

47 +-IR 8 2 * 715

45 | +-PETA ? < 11

46 | +-DEE GEO WOO GEN 2 2

73 | >DEE GEO WOO GEN ? # 2285932

48 +-IR 407 0 585

74 +-PETA ? * 11

75 +-IR 283 0 < 392

NAME - ">" indicates germplasm derived from germplasm in the line above

"+" indicates generative progenitor of the germplasm above the "+"

female progenitors are above male progenitors

DSTEP - Number of derivative steps from generative source

TERM - Termination code:

"*" indicates lines expanded elsewhere in the tree

"<" indicates lines which could be further expanded

"#" indicates germplasm with unknown parents
