# Supplementary figures and images for: Unraveling regulatory divergence, heterotic malleability, and allelic imbalance switching in rice due to drought stress
Source: Sci Rep. 2021 Jun 29;11:13489. doi: 10.1038/s41598-021-92938-x (PMC8241847; doi:10.1038/s41598-021-92938-x)

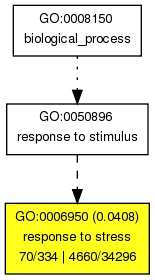

Supplement: Supplementary file 3 — Supplementary Information 3. [file 41598_2021_92938_MOESM3_ESM.jpg]

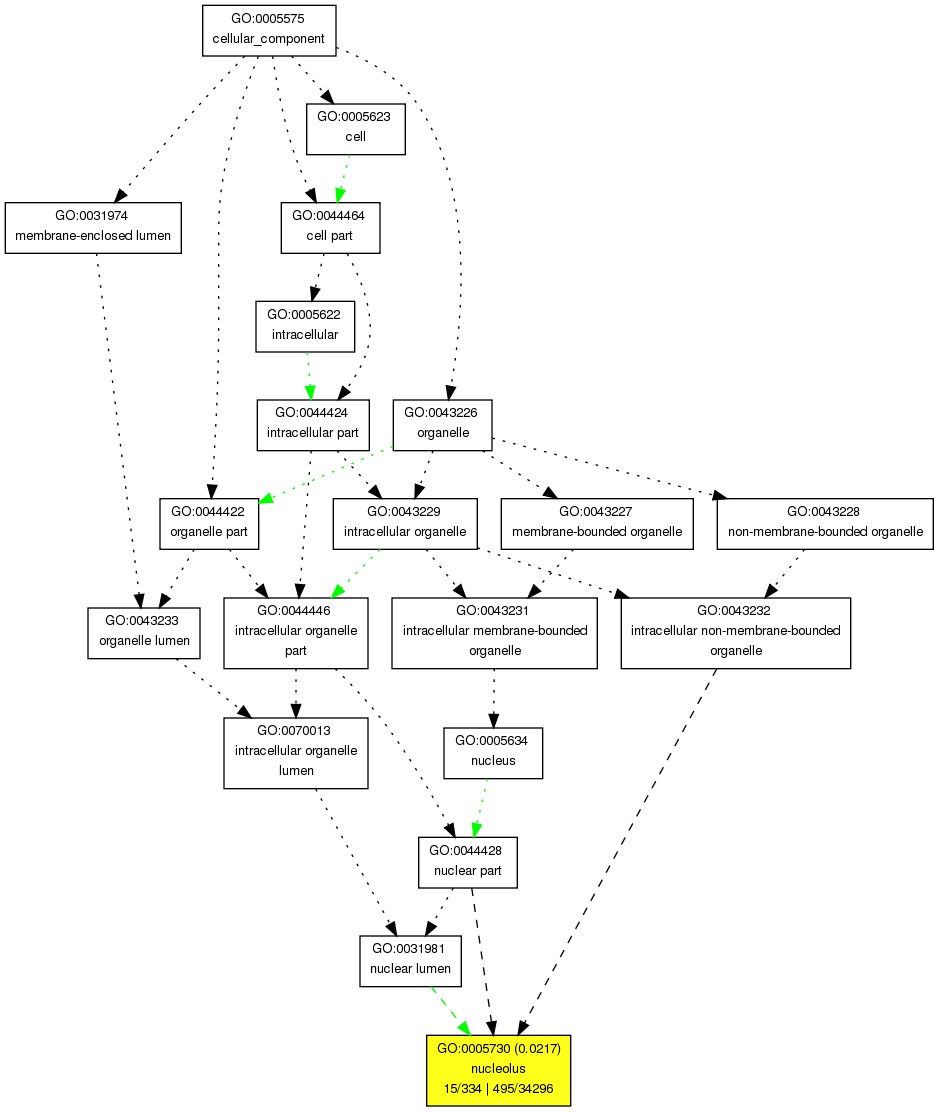

Supplement: Supplementary file 4 — Supplementary Information 4. [file 41598_2021_92938_MOESM4_ESM.jpg]

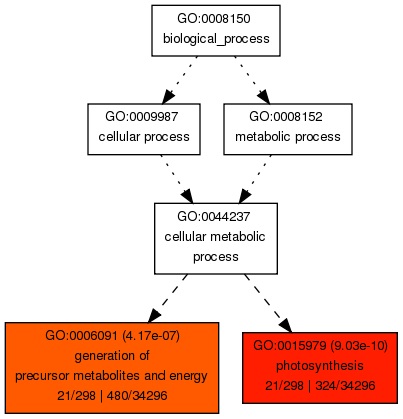

Supplement: Supplementary file 5 — Supplementary Information 5. [file 41598_2021_92938_MOESM5_ESM.jpg]

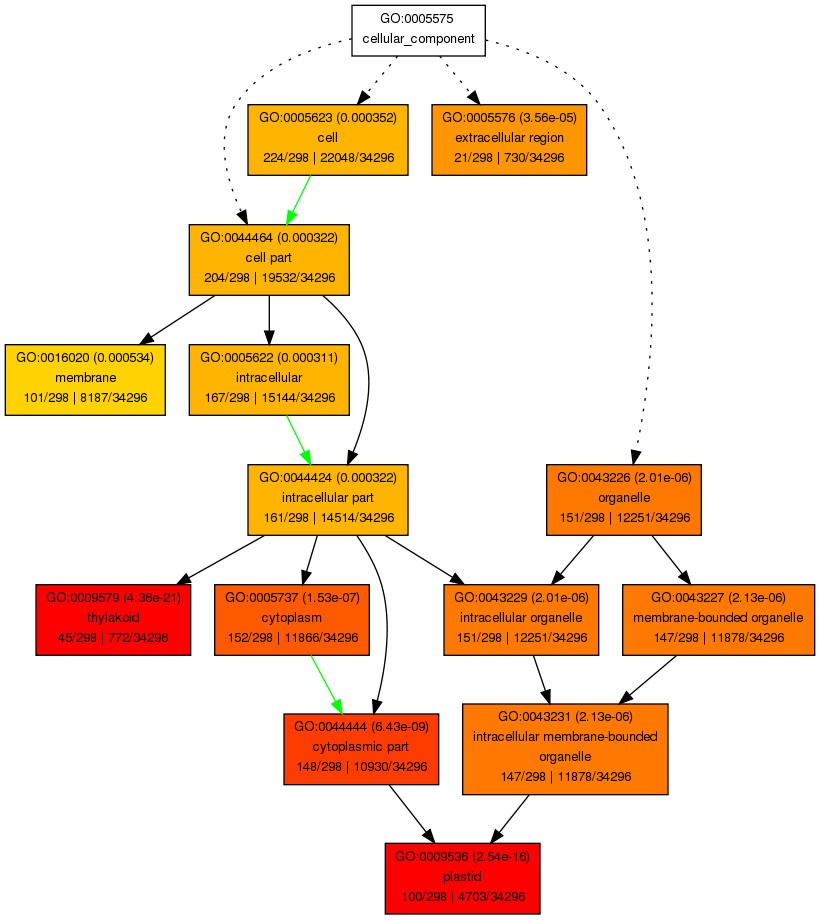

Supplement: Supplementary file 6 — Supplementary Information 6. [file 41598_2021_92938_MOESM6_ESM.jpg]

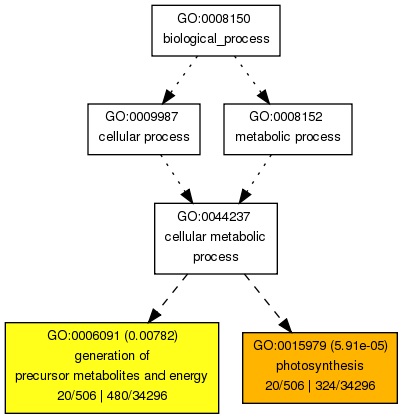

Supplement: Supplementary file 8 — Supplementary Information 8. [file 41598_2021_92938_MOESM8_ESM.jpg]

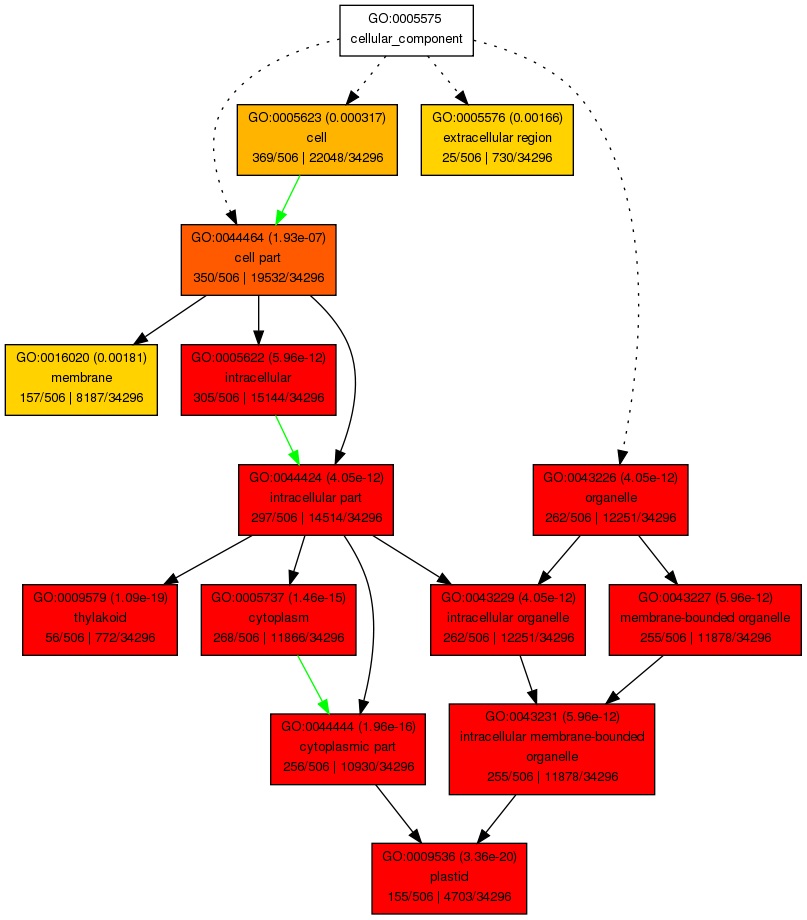

Supplement: Supplementary file 9 — Supplementary Information 9. [file 41598_2021_92938_MOESM9_ESM.jpg]

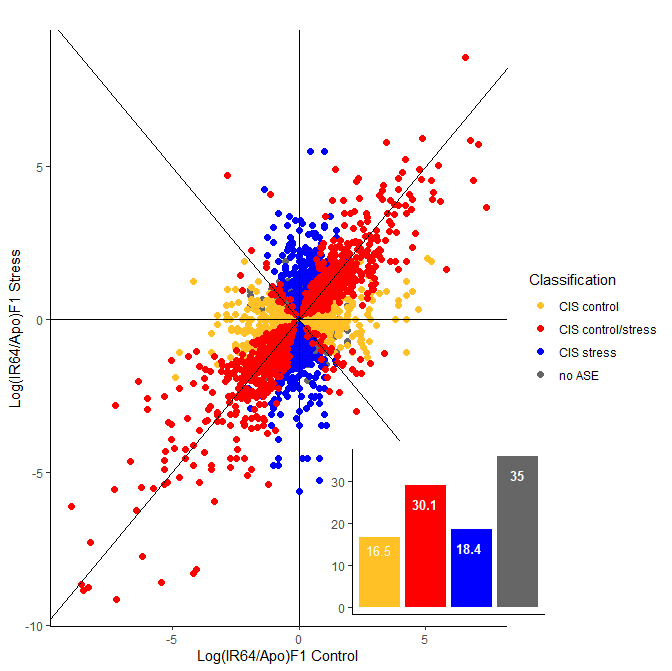

Supplement: Supplementary file 10 — Supplementary Information 10. [file 41598_2021_92938_MOESM10_ESM.png]

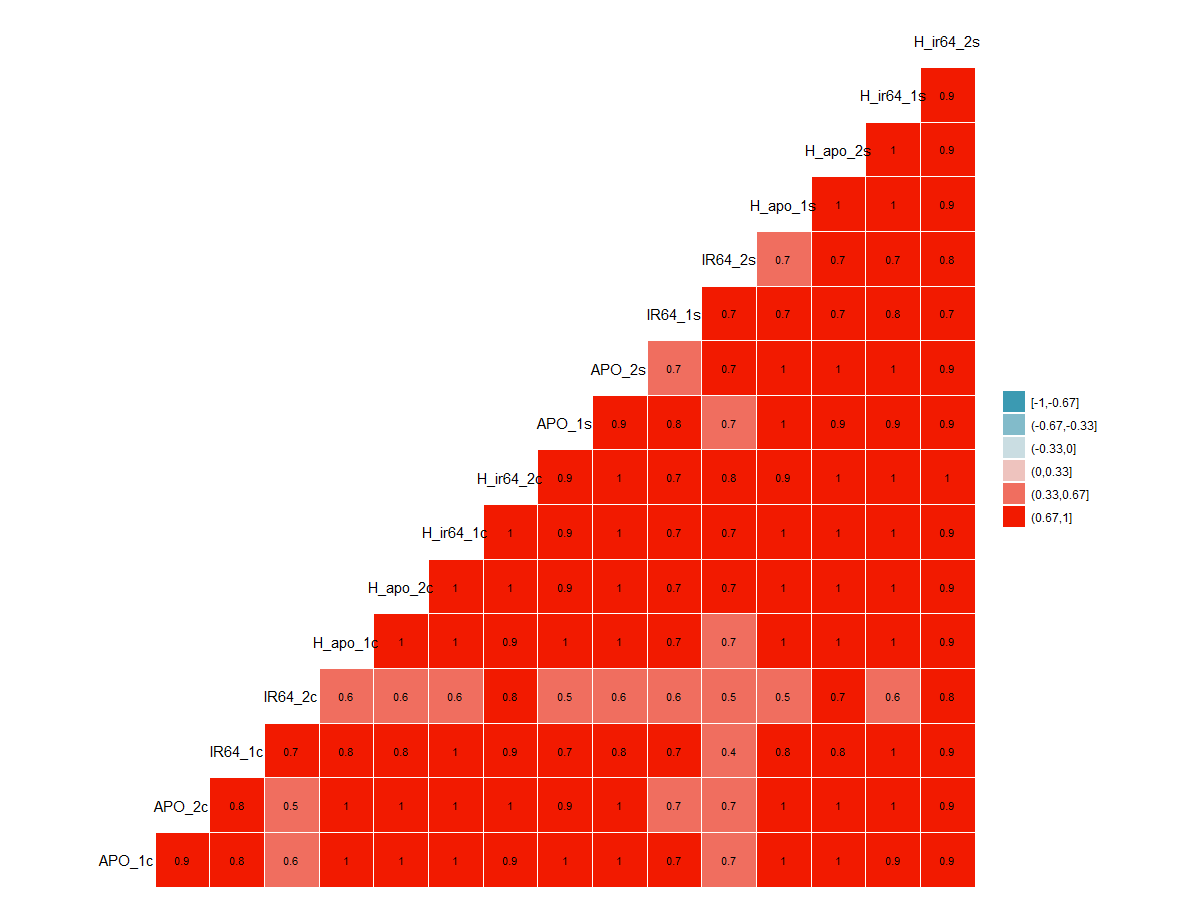

Supplement: Supplementary file 11 — Supplementary Information 11. [file 41598_2021_92938_MOESM11_ESM.png]
